# Advanced glycation end product levels were correlated with inflammation and carotid atherosclerosis in type 2 diabetes patients

**DOI:** 10.1515/biol-2020-0042

**Published:** 2020-06-11

**Authors:** Jie Li, Haiyan Shangguan, Xiaoqian Chen, Xiao Ye, Bin Zhong, Pen Chen, Yamei Wang, Bin Xin, Yan Bi, Dalong Zhu

**Affiliations:** Department of Endocrinology, Drum Tower Hospital Clinical College of Nanjing Medical University, Nanjing 210008, China; Department of Endocrinology, Nanjing Central Hospital, Nanjing 210008, China; Department of Endocrinology, Zhejiang Provincial People’s Hospital, Hangzhou 310000, China; Department of Endocrinology, People’s Hospital of Hangzhou Medical College, Hangzhou 310000, China; Department of Endocrinology, Drum Tower Hospital Affiliated to Nanjing University Medical School, Nanjing 210008, Jiangsu, China

**Keywords:** AGEs, carotid atherosclerosis, RAGE, T2DM, TNF-α, IFN-γ

## Abstract

Diabetes mellitus with atherosclerosis (AS) adds to the social burden. This study aimed to investigate whether advanced glycation end product (AGE) levels were correlated with inflammation and carotid AS (CAS) in type 2 diabetes mellitus (T2DM) patients. A total of 50 elderly T2DM patients and 50 age-matched senior healthy subjects were recruited in this study. T2DM patients were classified into two groups based on the intima–media thickness (IMT) of the carotid artery from color Doppler ultrasonography. Patients with IMT > 1 mm were classified into the T2DM + CAS group (*n* = 28), and patients with IMT < 1 mm were assigned as the T2DM + non-atherosclerosis (NAS) group (*n* = 22). The plasma levels of AGEs, receptor for AGE (RAGE), tumor necrosis factor alpha (TNF-α), and interferon gamma (IFN-γ) of all subjects were measured by enzyme-linked immunosorbent assay. The T-lymphocyte subsets were analyzed by a flow detector. T2DM + CAS patients showed significantly higher concentrations of AGEs, RAGE, TNF-α, and IFN-γ in the peripheral blood. The highest levels of CD4+ T cells were observed in the T2DM + CAS group. The AGE level was positively correlated with the concentrations of RAGE, TNF-α, IFN-γ, and CD4+. In summary, the results showed that the levels of AGEs may be correlated with the inflammatory status in T2DM patients with CAS.

## 1 Introduction

Diabetes mellitus is a metabolic disorder characterized by the impaired response to insulin and decreased insulin secretion in the body, resulting in chronic hyperglycemia [1]. Type 2 diabetes mellitus (T2DM) is the most common type of diabetes with a global prevalence of 350 million people in 2014 [2]. The number of T2DM patients is expected to increase significantly in the coming years, posing serious health and economic challenges. T2DM is associated with many long-term complications in the heart, blood vessels, kidney, nerves, and eyes. About 4.9 million people die of diabetes each year, in which around 50% is due to cardiovascular complications [3]. Atherosclerosis (AS) is a common complication of T2DM. Early detection and intervention of AS are of vital significance in the comprehensive management of T2DM. Carotid artery (CA) is the most easily involved blood vessel in AS; and its location is relatively superficial, but the degree and nature of carotid AS (CAS) can reflect the severity of the lesion. AS is actually a chronic inflammatory disease, in which various inflammatory and immune responses are involved. It has been reported that patients with diabetes presented larger necrotic cores in their coronary arteries and enhanced inflammation involved with macrophages and T-lymphocytes compared to patients without diabetes [4]. The intima–media thickness (IMT) of CA, defined as the distance from the leading edge of the media–adventitia interface to the leading edge of the lumen–intima interface, has been widely used as an indicator of the level of AS development [5,6].

Increased serum level of advanced glycation end products (AGEs) has been reported in T2DM patients with AS, which suggests its association with the development of vascular complications [7]. AGEs can interact with the receptor for AGEs (RAGEs) to induce inflammation [8,9]. Late glycosylation can induce glycosylation of proteins related to lipid metabolism, leading to lipid function damage and lipid metabolism disorder and eventually vascular complications in diabetes [10,11]. There are different degrees of lymphocyte infiltration in human atherosclerotic plaques. The T cells also play an important role in AS at the early stage, especially the clonal selection and expansion of their subsets [12]. Lymphocyte subsets mainly participate in the formation of AS by secreting cytokines [13,14].

To further understand how AGEs accelerate diabetic AS, we investigated the plasma level of AGEs, RAGE, T-lymphocyte subsets, and inflammatory cytokines including tumor necrosis factor alpha (TNF-α) and interferon gamma (IFN-γ) in the peripheral blood of elderly patients with type 2 diabetes. This study was undertaken to observe the effects of AGEs on T-lymphocyte-secreting inflammatory cytokines and to provide new insights for the prevention and treatment of complications of type 2 diabetes.

## 2 Materials and methods

### 2.1 Participants

Fifty patients with T2DM (*n* = 50, male/female: 21/29) were recruited in this study. T2DM was diagnosed based on 1999 World Health Organization criteria: fasting plasma glucose ≥7.0 mmol/L or 2-h plasma glucose ≥11.1 mmol/L. The inclusion criteria were (1) newly diagnosed T2DM patients, from January to August 2018; (2) consistent diet and/or treatment plans for 2 weeks; (3) age between 60 and 70 years; (4) body mass index between 19 and 35 kg/m^2^; and (5) no symptoms of diabetic ketoacidosis observed in the past 6 months. Patients were excluded according to the following criteria: (1) type 1 diabetes mellitus; (2) clinical signs of acute and/or chronic infection; tumor, hematologic diseases, liver disease, renal dysfunction, cardiovascular, and cerebrovascular diseases; and (3) history of smoking. Fifty healthy elderly participants were also included in this study (*n* = 50; male/female: 26/24). All healthy subjects showed normal fasting plasma glucose (<6.1 mmol/L), normal 2-h plasma glucose (<7.8 mmol/L), and had no sign of CAS. All the clinical parameters of the recruited subjects are shown in Table A1.


**Informed consent:** Informed consent has been obtained from all individuals included in this study.
**Ethical approval:** The research related to human use has been complied with all the relevant national regulations, institutional policies and in accordance with the tenets of the Helsinki Declaration, and has been approved by the Ethics Committee of Clinical Research.

### 2.2 CA ultrasonography

Color Doppler ultrasonography was performed to measure the IMT of CA by using an ultrasound scanner (HP5500; GE, USA) with a linear transducer of 5–10 MHz frequency. Patients were examined at a supine position. The B-mode gray scale images of the common CA, the bulb, the internal carotids, and the external carotids were recorded. The IMT measures the thickness of the two layers of the artery wall, tunica intima, and tunica media. The normal IMT of CA as evaluated by B-mode imaging is less than 1.0 mm. IMT at or above 1 mm is considered to be associated with AS. Based on the results from CA ultrasonography, 50 diabetes patients were divided into two subgroups: (1) T2DM + NAS group (*n* = 22; male/female: 10/12) with IMT < 1.0 mm and (2) T2DM + CAS group (*n* = 28; male/female: 11/17) with IMT ≥ 1.0 mm.

### 2.3 Enzyme-linked immunosorbent assay (ELISA)

Peripheral blood was taken from all subjects following 8 h of fasting. The levels of AGEs, RAGE, TNF-α, and IFN-γ in the peripheral blood were measured by AGEs, RAGE, TNF-α, and IFN-γ ELISA kits (Uscn life, USA), respectively, according to the manufacturer’s protocol.

### 2.4 T-lymphocyte subset analysis

Fasting peripheral whole blood was collected between 7:00 and 9:00 am. Heparinized peripheral whole blood (400 µL) was diluted into 400 µL RPMI1640 medium, added with 42 µL 1 µg/mL of phorbol myristate acetate, 33 µL 50 µg/mL of ionomycin, and 13.6 µL 0.1 mg/mL monensin (Sigma, Saint Louis, USA) and incubated at 37°C/5% CO_2_ for 4.5 h. Then the peripheral blood mononuclear cells (PBMCs) were separated by high-speed refrigerated centrifuge (Eppendorf, Germany). For staining, 100 µL of PBMCs was incubated with PerCP-Cy5.5 conjugated antihuman CD4 monoclonal antibody (clone: OKT4, Cat.: 85-45-0048-42) and antihuman CD25 monoclonal antibodies (clone: BC96, Cat.: 85-17-0259-42), separately. Each lymphocyte subset was collected by a flow cytometer (BD, USA), and the data were analyzed by FlowJo.

### 2.5 Statistical analysis

Software SPSS (Version 21.0) was used for statistical analysis. All data are reported as mean ± standard deviation and all data are normally distributed. Significant difference between the mean values of two groups was calculated by *t* test. One-way analysis of variance was used when comparison was among more than two groups. Correlation between AGEs and other clinical indicators was determined by age-adjusted partial correlation coefficient analysis. *p* < 0.05 was considered to be statistically significant.

## 3 Results

### 3.1 T2DM patients with CAS showed significantly higher IMT and elevated plasma levels of AGEs and RAGE

**Figure 1:**
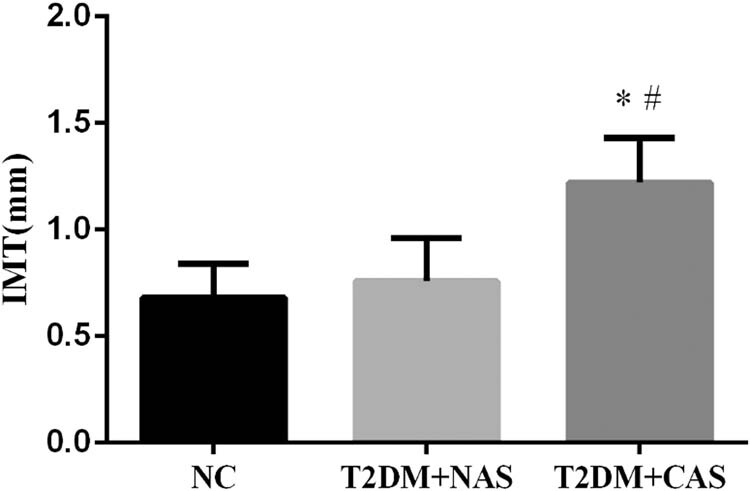
IMT of CA in healthy subjects, T2DM + NAS group, and T2DM + CAS group. The IMT of CA was examined by color Doppler ultrasonography. The IMT value of less than 1.0 mm was considered as normal. * indicates statistical significance (*p* < 0.05) compared to healthy subjects (NC); # indicates statistical significance (*p* < 0.05) compared to T2DM + NAS group.

**Figure 2:**
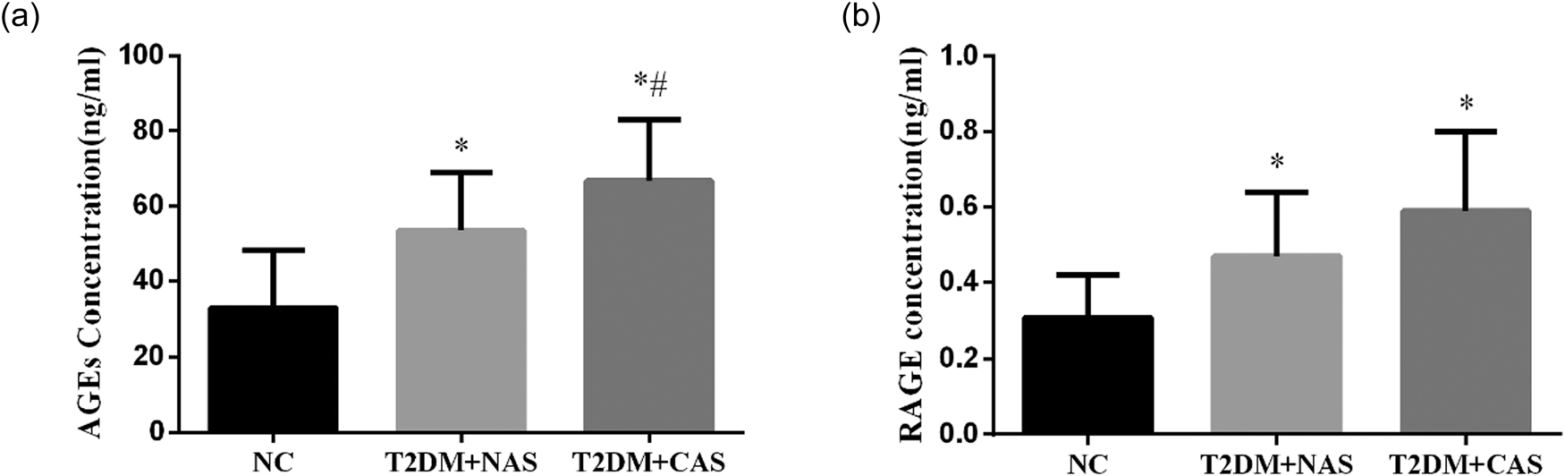
Plasma levels of AGEs and RAGE in healthy subjects, T2DM + NAS group, and T2DM + CAS group. Expression levels of AGEs (a) and RAGE (b) in peripheral blood were tested by ELISA in all three groups. * indicates statistical significance (*p* < 0.05) compared to healthy subjects (NC); # indicates statistical significance (*p* < 0.05) compared to the T2DM + NAS group.

The IMT of CA in the healthy subjects was 0.68 ± 0.16 mm, which was not significantly different from that in the T2DM + NAS group (0.76 ± 0.20 mm). However, in the T2DM + CAS group, their average IMT (1.22 ± 0.21 mm) was significantly higher compared to that in the healthy subjects and the T2DM + NAS group (Figure 1). Plasma levels of AGEs in healthy subjects, T2DM + NAS group, and T2DM + CAS group were 32.85 ± 15.26, 53.47 ± 15.39, and 66.71 ± 16.36 ng/mL, respectively (Figure 2a). Healthy subjects showed significantly lower level of AGEs compared to the other two groups. Plasma level of RAGE was also significantly lower in the control group (0.31 ± 0.11 ng/mL) than that in the T2DM + NAS (0.47 ± 0.17 ng/mL) and T2DM + CAS groups (0.59 ± 0.21 ng/mL; Figure 2b).

### 3.2 Increased expression levels of TNF-α and IFN-γ were observed in T2DM patients with and without CAS

**Figure 3:**
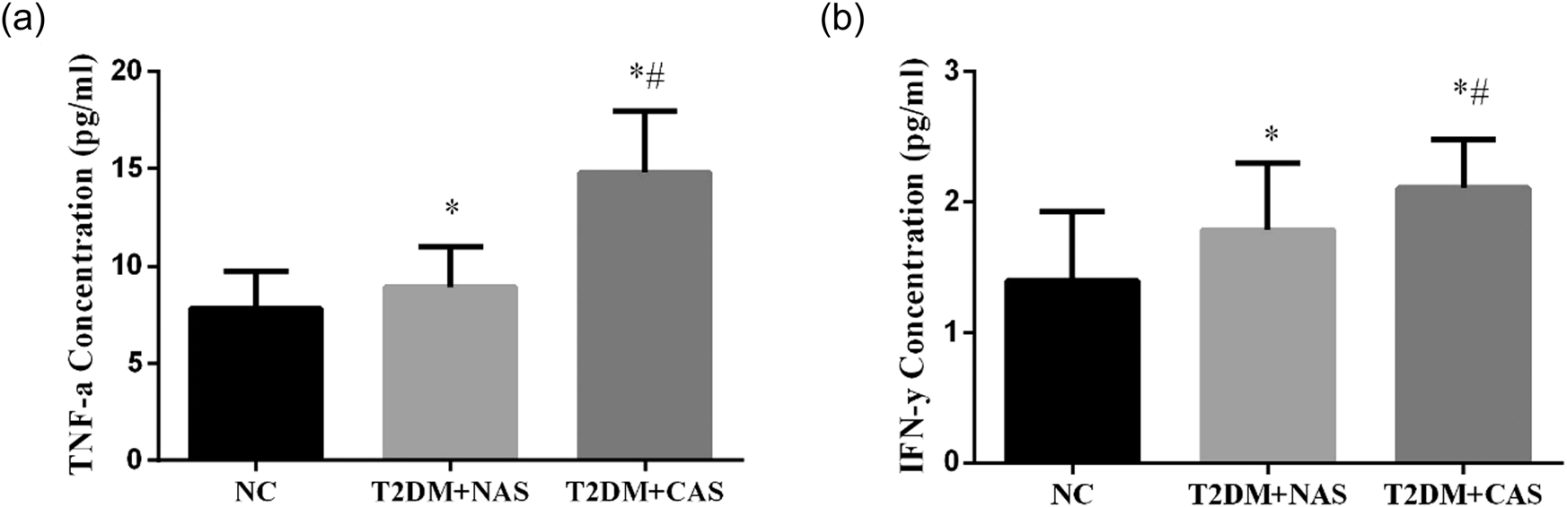
Expressions levels of TNF-α and IFN-γ in healthy subjects, T2DM + NAS group, and T2DM + CAS group. Expression levels of TNF-α (a) and IFN-γ (b) in peripheral blood were tested by ELISA in all three groups. * indicates statistical significance (*p* < 0.05) compared to healthy subjects (NC); # indicates statistical significance (*p* < 0.05) compared to the T2DM + NAS group.

The expressions of inflammatory cytokines including TNF-α and IFN-γ were significantly higher in the T2DM + NAS and T2DM + CAS groups than those in the control group. The plasma levels of TNF-α were 7.82 ± 1.92, 8.94 ± 2.05, and 14.79 ± 3.15 pg/mL in healthy subjects, T2DM + NAS group, and T2DM + CAS group, respectively (Figure 3a). Meanwhile, the levels of IFN-γ were 1.40 ± 0.53, 1.61 ± 0.51, and 2.11 ± 0.37 pg/mL in the respective three groups (Figure 3b).

### 3.3 The number of CD4+ T cells was increased in T2DM + CAS patients

Both the T2DM + NAS and T2DM + CAS groups had a significantly larger amount of CD4+ T cells compared to healthy subjects. Moreover, the number of CD4+ T cells in T2DM + NAS patients was significantly higher than that in the T2DM + CAS patients ( *p* < 0.05). The ratio of CD4+/CD8+ was also significantly lower in the healthy groups. In contrast, T2DM + CAS showed the least amount of CD8+ T cells among these groups (Table 1).

**Table 1:** Determination of T-cell subsets in peripheral blood

Grouping	CD4+ (%)	CD8+ (%)	CD4+/CD8+ (%)	CD4+ CD25+ Treg (%)
Control	20.58 ± 1.46	26.34 ± 1.99	0.88 ± 0.12	4.10 ± 0.61
T2DM + NAS	23.49 ± 2.11^a^	24.16 ± 2.23^a^	0.97 ± 0.15^a^	3.77 ± 0.35^a^
T2DM + CAS	31.37 ± 2.49a,b	23.05 ± 2.51^a^	1.35 ± 0.24a,b	3.36 ± 0.34a,b

a *p* < 0.05 compared with the normal control group.

b *p* < 0.05 compared with the T2DM + NAS group.

### 3.4 Correlation analysis of AGEs and other clinical indicators

**Figure 4:**
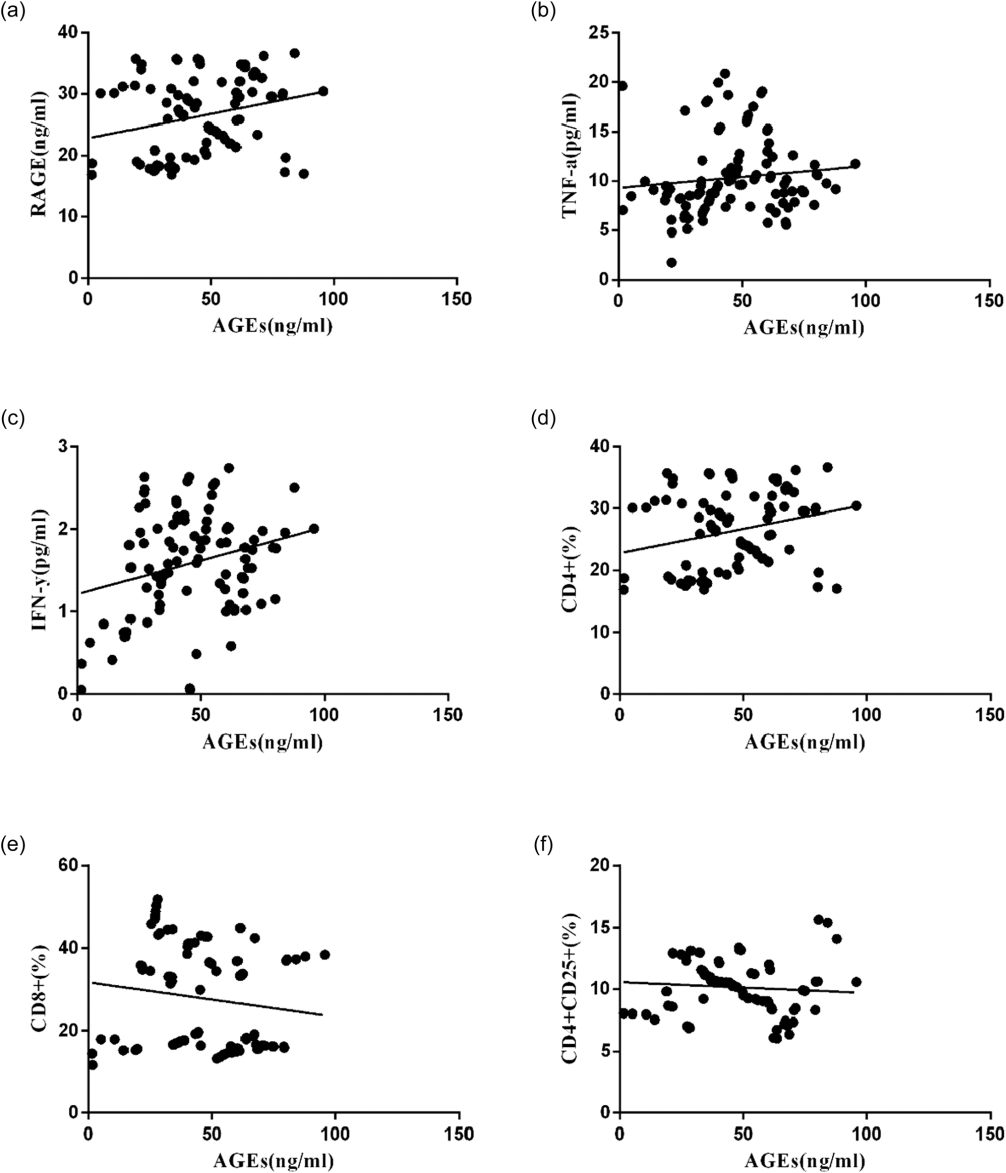
Scatter plots of linear correlation between peripheral blood AGE level and other clinical indicators. Pearson’s correlation coefficient analysis was used to calculate the correlation between peripheral blood AGEs level and RAGE (a), TNF-α (b), IFN-γ (c), CD4+ (d), CD8+ (e), and CD4+ CD25+ (f).

Correlation analysis was performed and the results showed that there was a positive correlation between AGEs and RAGE (Figure 4a), TNF-α (Figure 4b), IFN-γ (Figure 4c), and CD4+ (Figure 4d; *P* < 0.05). However, it was negatively correlated with CD8+ (Figure 4e) and CD4+ CD25+ (Figure 4f and Table 2).

**Table 2:** Correlation analysis of AGEs and other clinical indicators in three groups

Index	Partial correlation analysis	
	*r*	*p*
RAGE (ng/ml)	0.4388	0.0074
TNF-α (pg/ml)	0.2461	0.0189
IFN-γ (pg/ml)	0.1745	0.0301
CD4+ (%)	0.2503	0.0065
CD8+ (%)	−0.1566	0.0797
CD4+ CD25+ (%)	−0.1743	0.0612

## 4 Discussion

AS is a common vascular complication in T2DM [15]. IMT has been widely used as a noninvasive indicator of AS at subclinical stage [16]. Our lab performed color Doppler ultrasonography to measure the IMT of CA in senior T2DM patients. The diagnostic criterion of AS is IMT ≥ 1 mm. Based on the results of IMT, we classified 50 T2DM patients into the T2DM + CAS group (*n* = 22) with IMT > 1 mm and the T2DM + NAS group (*n* = 28) with IMT < 1 mm.

AGEs can be rapidly cleaned up from blood [17], and the levels of AGEs were low in normal physiological conditions. However, aging or diabetes with high sugar content can accelerate the saccharification process, leading the body to produce AGEs spontaneously. When the generation rate is higher than the degradation rate, it will lead to accumulation of AGEs in the body [18]. AGEs interact with their RAGE to initiate intracellular signaling pathways involving the change in vascular structure and then accelerate the progression of AS [19]. RAGE is a multiligand receptor that belongs to the immunoglobulin superfamily, often seen on the surface of smooth muscle cells, macrophages, T-lymphocytes, glomerular podocytes, and neurons [20]. Most studies focused on the effect of AGE-RAGE on reactive oxygen species generation in the process of AS. However, whether AGEs-RAGE interaction causes AS by affecting T lymphocytes remains unknown.

AS is characterized by chronic inflammation. Numerous neutrophils, mononuclear macrophages, and T lymphocytes are found in plaques at various stages of AS, so the relationship between AS and innate immune pattern recognition has been of concern for a long time. Recent studies suggest that AGEs are a molecular pattern related to damage in the body, and RAGE is a pattern recognition receptor. After being recognized by RAGE, AGEs can activate the immune response. From the perspective of immune mechanism, the research on the mechanism of AGE-RAGE reaction promoting the occurrence and development of diabetic AS is a hot spot.

Previously, it has been found that T cells express the receptor of AGEs on the surface, and RAGE with a molecular weight of 50–60 kd is the main one. Imani et al. labeled AGEs with I125 to observe the binding ability of peripheral lymphocyte surface receptors [21]. It was found that the ability of lymphocyte-binding AGEs significantly increased after 48 h of pre-stimulation of peripheral blood lymphocytes by phytohemagglutinin. CD4+ T cells increased from 34.2% to 92%, while CD8+ T cells increased from 58.5% to 90%, and it was found that peripheral blood lymphocytes expressed IFN-γ up to ten times higher. This suggests that AGE is one of the factors affecting immune disorders of T cells and plays a role in vascular complications of diabetes mellitus.

The major class of T lymphocytes present in AS is CD4+, which can differentiate into Th1 and Th2 cells based on the local milieu of cytokines. The Th1 lineage may be the key regulator of lymphocytic influence in the development of AS [22]. Both Th1 cells and CD8+ T cells can secrete inflammatory cytokines IFN-γ and TNF-α. We found significantly higher levels of AGEs, RAGE, IFN-γ, and TNF-α in the T2DM + CAS group compared with the T2DM + NAS group and controls. It suggested that the increased concentration of AGEs and RAGE in peripheral blood was correlated with the occurrence and development of AS in T2DM. Meanwhile, T2DM + CAS patients also showed imbalanced T cells and decreased Treg cells. Pearson analysis revealed a positive correlation between AGEs and RAGE, CD4+, IFN-γ, and TNF-α, suggesting that combining AGEs and RAGE activates the immune response in the body and promotes the secretion of inflammatory factors IFN-γ and TNF-α. Weiser et al. showed that IFN-γ synergized with other cytokines to elevate the expression of the adhesion molecules, vascular cell adhesion molecule-1 in brain endothelial cells [23]. IFN-γ also impaired the cellular cholesterol balance, by reducing the expression of cholesterol 27-hydroxylase, to facilitate the pathogenesis of AS [24]. Another potential mechanism of IFN-γ contributing to the atherosclerotic process was through a p53-dependent DNA damage pathway in cellular senescence [25]. TNF-α is also considered as a key factor involved in AS. Ridker et al. found that the plasma concentration of TNF-α is persistently increased in postmyocardial ischemia patients with higher risk of recurrent coronary events [26]. Treg cells were also reported to have a regulatory effect on the initiation and progression of AS [27]. In this study, senior T2DM + CAS patients showed a lower concentration of CD4+ CD25+ Treg in peripheral blood than that in the healthy subjects. Although no correlation was found between AGEs vs CD8+ and AGEs vs Treg, significantly decreased levels of CD8+ T cells and Treg cells were observed in T2DM + CAS patients. It suggests the involvement of CD8+ T cells and Treg cells in AS.

In this study, we would like to address several study limitations. First, the study was performed in a single center, and further studies may involve multicenter so as to increase the power of our current findings. Second, as using IMT as a predictor of cardiovascular risk has been controversial among studies [28], we should carefully interpret our findings and may employ more diagnostic tools to confirm the current findings. Third, conflicting results regarding the role of AGEs in the development of coronary artery disease have been reported [29]; further studies may increase the sample size to confirm our findings. Finally, the present study suggested a link between AGEs and the extent of inflammation; however, whether the changes in the inflammatory status contribute to the development of coronary artery disease in T2DM patients still require mechanistic studies.

In conclusion, the development of AS in T2DM is a complex process. In the present study, our results showed that the levels of AGEs may be correlated with the inflammatory status in T2DM patients with CAS. Our results may suggest a link between AGE levels and the extent of inflammation, which may contribute to the development of CAS in T2DM patients.

## Appendix

**Table A1:** Clinical parameters of the recruited subjects

Parameters	NC	T2DM	*P*
Age (years)	65.6 ± 3.1	64.7 ± 3.9	>0.05
BMI (kg/m^2^)	24.3 ± 2.6	27.6 ± 3.2	<0.05
Triglycerides (mg/dL)	111.7 ± 42.3	123.7 ± 35.6	>0.05
Total cholesterol (mg/dL)	163.7 ± 32.3	156.5 ± 38.9	>0.05
HDL cholesterol (mg/dL)	41.2 ± 9.9	44.1 ± 11.4	>0.05
LDL cholesterol (mg/dL)	104.8 ± 28.9	94.9 ± 21.7	>0.05
Glycaemia (mg/dL)	92.6 ± 10.2	151.4 ± 21.1	<0.05
HbA1c (%)	6.1 ± 0.4	7.4 ± 0.7	<0.05
